# Clinical and molecular surveillance of artemisinin resistant falciparum malaria in Myanmar (2009–2013)

**DOI:** 10.1186/s12936-017-1983-9

**Published:** 2017-08-14

**Authors:** Myat Htut Nyunt, Myat Thu Soe, Hla Win Myint, Htet Wai Oo, Moe Moe Aye, Soe Soe Han, Ni Ni Zaw, Cho Cho, Phyo Zaw Aung, Khin Thiri Kyaw, Thin Thin Aye, Naychi Aung San, Leonard Ortega, Krongthong Thimasarn, Maria Dorina G. Bustos, Sherwin Galit, Mohammad Rafiul Hoque, Pascal Ringwald, Eun-Taek Han, Myat Phone Kyaw

**Affiliations:** 1 Department of Medical Environmental Biology and Tropical Medicine, School of Medicine, Kangwon National University, Chuncheon, Gangwon-do Republic of Korea; 2 Department of Medical Research, Yangon, Republic of the Union of Myanmar; 3 Magway District Hospital, Ministry of Health and Sports, Magway, Republic of the Union of Myanmar; 4 World Health Organization, Geneva, Switzerland; 5 World Health Organization Country Office for Myanmar, Yangon, Republic of the Union of Myanmar; 6 World Health Organization Country Office for Thailand, Bangkok, Thailand; 7 Research Institute for Tropical Medicine, Alabang, Muntinlupa City, Philippines

**Keywords:** Drug resistance, Falciparum, Malaria, Myanmar, Kelch 13, Artemisinin

## Abstract

**Background:**

Emergence of artemisinin-resistant malaria in Southeast Asian countries threatens the global control of malaria. Although K13 kelch propeller has been assessed for artemisinin resistance molecular marker, most of the mutations need to be validated. In this study, artemisinin resistance was assessed by clinical and molecular analysis, including *k13* and recently reported markers, *pfarps10*, *pffd* and *pfmdr2*.

**Methods:**

A prospective cohort study in 1160 uncomplicated falciparum patients was conducted after treatment with artemisinin-based combination therapy (ACT), in 6 sentinel sites in Myanmar from 2009 to 2013. Therapeutic efficacy of ACT was assessed by longitudinal follow ups. Molecular markers analysis was done on all available day 0 samples.

**Results:**

True recrudescence treatment failures cases and day 3 parasite positivity were detected at only the southern Myanmar sites. Day 3 positive and *k13* mutants with higher prevalence of underlying genetic foci predisposing to become *k13* mutant were detected only in southern Myanmar since 2009 and comparatively fewer mutations of *pfarps10, pffd*, and *pfmdr2* were observed in western Myanmar. *K13* mutations, V127M of *pfarps10*, D193Y of *pffd*, and T448I of *pfmdr2* were significantly associated with day 3 positivity (OR: 6.48, 3.88, 2.88, and 2.52, respectively).

**Conclusions:**

Apart from *k13*, *pfarps10, pffd* and *pfmdr2* are also useful for molecular surveillance of artemisinin resistance especially where *k13* mutation has not been reported. Appropriate action to eliminate the resistant parasites and surveillance on artemisinin resistance should be strengthened in Myanmar.

*Trial registration* This study was registered with ClinicalTrials.gov, identifier NCT02792816.

## Background

Artemisinin-based combination therapy (ACT) is the only recommended mainstream treatment for falciparum malaria in almost all endemic countries [1]. Decreased susceptibility of artemisinin was first reported in Cambodia [2], followed by Thailand [3] and Myanmar [4]. As of 2015, artemisinin-resistant falciparum malaria has been documented in 5 of the Greater Mekong countries: Cambodia, Laos People’s Democratic Republic, Myanmar, Thailand, and Vietnam [5]. As drug-resistant malaria has emerged, therapeutic efficacy studies have been conducted in almost all the endemic countries, initiated by the World Health Organization (WHO) [6].

In Myanmar, artemether–lumefantrine, artesunate–mefloquine or dihydroartemisinin–piperaquine has been deployed for treatment of falciparum malaria since 2002 [7] and delayed parasite clearance after treatment with ACT has been observed since 2009 [5]. Although the trend of malaria morbidity and mortality has been decreasing in Myanmar, malaria is still one of the top priority diseases and endemic in 284 out of 330 townships, as of 2014 [8].

After discovery of K13 kelch propeller artemisinin resistance molecular marker, many studies showed the prevalence of *k13* mutations in falciparum-endemic countries, including Myanmar [9–11]. Although independent emergence of the *k13* mutation was suggested [12], artemisinin resistance-associated *k13* mutations were detected only in Southeast Asian countries [13] but most of the *k13* mutations have not yet validated. Evidence of independent emergence of artemisinin resistance in several areas in the Greater Mekong Sub-region led to a change in strategy from containment to elimination of multi-drug resistant falciparum in the region [14]. Previous cross-sectional *k13* surveys in Myanmar covered most of the country, except southern Myanmar, and indicated widespread *k13* mutations across the country with high prevalence of *k13* mutants in northwestern Myanmar near the border areas with India [10]. Another independent cohort study in Myanmar [9] showed a lack of day 3 parasite positivity and no artemisinin resistance-associated *k13* mutations in Chin State, next to the border with Bangladesh. At the same time, no *k13* mutation was reported in Bangladesh [15] and India [16].

In this study, the efficacy of first-line anti-malarials in 6 sentinel sites was assessed by clinical follow-up and molecular marker analysis to confirm and validate the observed *k13* mutations for delayed parasite clearance. Moreover, newly identified molecular markers from genome-wide association study (GWAS), such as *pfarps10* (*Plasmodium falciparum* apicoplast ribosomal protein S10), *pffd* (*P. falciparum* ferredoxin), and *pfmdr2* (*P. falciparum* multidrug resistance protein 2) [17] were assessed as to whether they were also useful molecular markers for surveillance of artemisinin resistance.

## Methods

### Study area

The 6 sentinel sites were selected for surveillance of malaria based on the criteria described by WHO [6] which include the following considerations: population density, an area with local transmission of malaria, near the border area or direly related with border area, area with high mobile/migrant population, feasibility to conduct the study. The study area covered southern Myanmar (Kawthaung) where reduced susceptibility was first reported [4], southeast (Thanbyuzayat and Myawaddy), southern part of central Myanmar (Shwegyin), western part of central Myanmar (Magway) and western border area (Rakhine).

### Study design and recruitment

For this prospective cohort study, uncomplicated falciparum malaria patients were recruited according to WHO standardized protocol [6] in 6 sentinel sites in Myanmar from 2009 to 2013 to assess the therapeutic efficacy and safety of ACT. Patients were eligible if they were aged between 2 and 65 years (except in Magway where only children under 15 years of age were recruited as the previous hospital data indicated that recurrent fever after ACT in children were not uncommon), uncomplicated falciparum mono-infection by microscopy, parasite density no more than 250,000 parasites per μL, and fever (axillary temperature ≥37.5 °C) or history of fever in previous 24 h. Exclusion criteria included severe malaria, mixed species infection, non-falciparum infections, presence of other febrile diseases, regular medications that interfere with anti-malarial pharmacokinetics, history of hypersensitivity to tested anti-malarials, pregnant or lactating mothers, or unable to follow up after treatment.

### Procedures

The method for surveillance of anti-malarial drug efficacy provided by WHO [6] was followed in this study. Briefly, active and passive case detection was done in the sentinel sites and if patients met the inclusion criteria, direct observed treatment with ACT was carried out. If a recruited patient vomited within 30 min after treatment, second dose was administered. A patient is enrolled and given initial treatment of ACT, then scheduled with 28 days follow-up for artemether–lumefantrine and with 42 days follow-up for dihydroartemisinin–piperaquine or artesunate–mefloquine. Peripheral blood smears and a dried blood spot sample from finger prick were taken at each of the follow ups. Clinical and blood film examination was carried out at days 0, 1, 2, 3, 7, 14, 21, 28, 35, and 42. If fever or any signs of malaria appeared within the observation period, blood film examination was carried out to exclude treatment failure. If treatment failure was observed within the observation period, an alternative ACT was prescribed.

### Microscopic examination

Thick and thin blood films for parasite count were obtained and examined at screening on day 0 to confirm inclusion/exclusion criteria. Thick blood films were also examined on each follow-up visit or on any other day if the patient spontaneously returned and parasitological re-assessment was required. Parasite density was calculated by counting the number of asexual parasites against a set number of white blood cells (WBCs), typically 200–300, in thick blood film, using a hand tally counter. Parasite density, expressed as the number of asexual parasites per µL of blood, was calculated by dividing the number of asexual parasites by the number of WBCs and then multiplying by an assumed WBC density (typically 6000 WBCs/µL). A blood slide was considered negative when the examination of 1000 WBCs did not reveal any asexual parasites.

### Genotyping of malaria parasite

All treatment failure cases were genotyped as described previously [18] to differentiate a recrudescence (same parasite strain) from a newly acquired infection (different parasite strain) by analysis of the *msp1, msp2* and *glurp* genes from the samples collected on day 0 and day of failure. Genotypic profiles of the pre- and post-parasite strains were compared.

### Artemisinin resistance molecular markers analysis

The molecular marker analysis on the available 550 samples of day 0 from 6 different sentinel sites was conducted. DNA was extracted from dried blood spots by QIAamp DNA Blood Mini Kit (Qiagen, Valencia, CA) according to manufacturer’s recommendation. Amplification of artemisinin resistance molecular markers, *k13* (PF3D7_1343700) and other genetic foci such as *pfarps10* (PF3D7_1460900.1), *pffd* (PF3D7_1318100) and *pfmdr2* (PF3D7_1447900) were carried out according to the procedure described previously [19, 20]. The sequences were aligned with that of 3D7 retrieved from *Plasmodium* database and deposited in GenBank (Accession Numbers: KX280647–KX280707).

### Statistical analysis

Sample size required in each therapeutic efficacy study site was calculated based on the estimated anticipated population proportion of clinical failure rate, 15%, and precision 10% with 95% confidence level; at least 49 cases per site would be needed for analysis. For clinical follow-up data, the proportions of treatment failure and adequate clinical and parasitological response (ACPR) on day 28 or day 42, and day 3 parasite positivity after ACT, were calculated. Chi square test or Fisher’s exact tests were used to analyse the categorical data and Mann–Whitney’s *U* test for quantitative data. The proportion of patients who showed day 3 parasite positivity and mutations of each target genes was compared, and odds ratios (OR) were calculated. Correlation was calculated between wild type alleles for target genes and day 3 parasite positivity in each of the study sites. A significance level of 0.05 was used for all statistical tests.

## Results

### Therapeutic efficacy of ACT

**Fig. 1:**
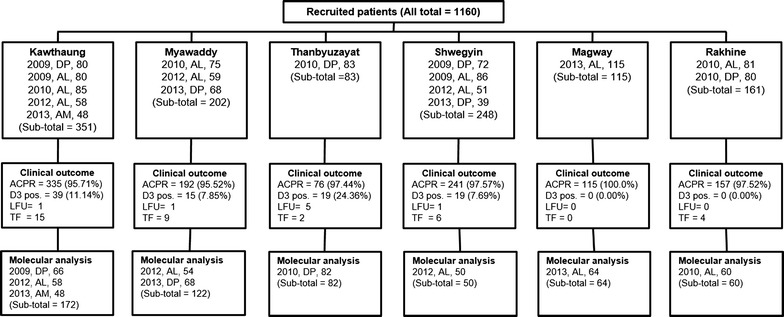
Summary of samples involved for clinical and molecular analysis. Year of the study, anti-malarial, and number of the samples collected are shown. *DP* dihydroartemisinin–piperaquine, *AL* artemether–lumefantrine, *AM* artesunate–mefloquine, *LFU* lost to follow-up, *TF* treatment failure, *ACPR* adequate clinical and parasitological response, *D3 pos.* day 3 parasite positivity

**Fig. 2:**
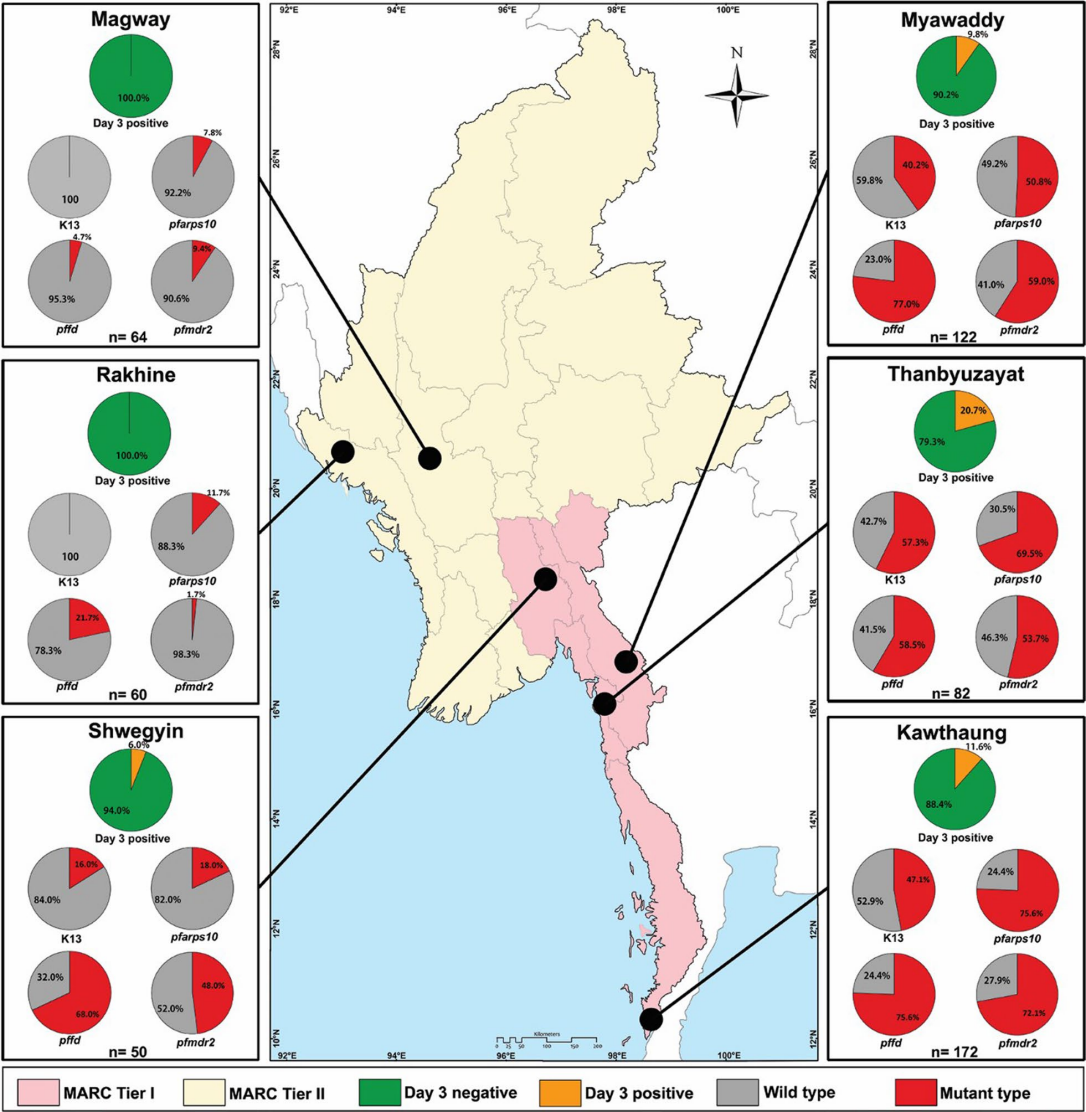
Distribution of day 3 positivity after treatment with artemisinin-based combination therapy (ACT) and molecular markers (*k13*, *pfarps10*, *pffd* and *pfmdr2*) in six sentinel sites. Day 3 prevalence and high mutant rate of molecular markers were observed in southern Myanmar sites, Myanmar Artemisinin Resistance Containment (MARC) Tier I areas

The therapeutic efficacy study of the first-line anti-malarial in different sentinel sites (Table 1; Fig. 1) was done. A total of 1160 from 6 sentinel sites were recruited with longitudinal follow-up according to WHO standardized protocol [6] to understand the clinical and parasitological response to anti-malarials. Overall, ACPR was 1116/1152 (96.87%) and lowest rate was noted in Kawthaung, southern Myanmar site 79/84 (94.0%) in 2010 after treatment with artemether–lumefantrine. Four out of 16 studies showed 100% ACPR, which included Kawthaung (southern Myanmar) after treatment with artemether–lumefantrine in 2012, artesunate-mefloquine in 2013, Shwegyin (southern part of central Myanmar) after treatment with dihydroartemisinin–piperaquine in 2009, and Magway (western part of central Myanmar) after treatment with artemether–lumefantrine in 2013. Day 3 parasite positivity rate was 8.0% with geometric mean of 260 parasites/μL (median: 225 parasites/μL, range 11–13,205 parasites/μL,) and highest in Thanbyuzayat after treatment with dihydroartemisinin–piperaquine in 2010 (24.4%) (Table 1; Fig. 2). There was no day 3 parasite positivity in Magway and Rakhine, western border to Bangladesh. Except two cases of early treatment failure (fever with day 3 parasite positivity) in Shwegyin, all others were late treatment failures.

**Table 1:** Summary result of therapeutic efficacy study in six sentinel sites in Myanmar (2009–2013)

Description	Kawthaung					Myawaddy			TBZ	Shwegyin				Rakhine		Magway
Year	2009	2009	2010	2012	2013	2010	2012	2013	2010	2009	2009	2012	2013	2010	2010	2012
Drug	DP	AL	AL	AL	AM	AL	AL	DP	DP	DP	AL	AL	DP	AL	DP	AL
Recruited cases	80	80	85	58	48	75	59	68	83	72	86	51	39	81	80	115
Treatment failure	4	6	5	0	0	4	2	3	2	0	2	3	1	3	1	0
LFU	0	0	1	0	0	1	0	0	5	0	0	1	0	0	0	0
Day 1 parasite positivity (n, %)	76 (95.0)	52 (65.0)	75 (89.3)	47 (81.0)	40 (83.3)	61 (82.4)	39 (66.1)	52 (76.5)	76 (97.4)	51 (70.8)	72 (83.7)	9 (18.0)	11 (28.2)	31 (38.3)	39 (48.8)	81 (70.4)
Day 2 parasite positivity (n, %)	54 (67.5)	30 (37.5)	38 (45.2)	12 (20.7)	23 (47.9)	22 (29.7)	15 (25.4)	16 (23.5)	44 (56.4)	15 (20.8)	30 (34.9)	2 (4.0)	1 (2.6)	1 (1.2)	3 (3.8)	31 (27.0)
Day 3 parasite positivity (n, %)	15 (18.7)	5 (6.3)	7 (8.3)	7 (12.1)	5 (10.4)	3 (4.1)	8 (13.6)	4 (5.9)	17 (20.5)	3 (4.2)	8 (9.3)	3 (6.0)	5 (12.8)	0 (0.0)	0 (0.0)	0 (0.0)
ACPR (n, %)	76 (95.0)	74 (92.5)	79 (94.0)	58 (100.0)	48 (100.0)	70 (94.6)	57 (96.6)	65 (95.6)	76 (97.4)	72 (100.0)	84 (97.7)	47 (94.0)	38 (97.4)	78 (96.3)	79 (98.7)	115 (100.0)
Recrudescence	2	6	2	0	0	1	2	3	1	0	0	1	0	0	0	0
Reinfection	1	0	2	0	0	2	0	0	1	0	1	1	1	3	1	0

*TBZ* Thanbyuzayat, *DP* dihydroartemisinin–piperaquine, *AL* artemether–lumefantrine, *LFU* loss to follow-up, *ACPR* adequate clinical and parasitological response, *ETF* early treatment failure, *LCF* late clinical failure, *LPF* late parasitological failure

### Parasite genotyping

All treatment failure cases were genotyped using *msp*-*1*, *msp*-*2* and *glurp* gene analysis from the paired samples collected on day 0 and day of failure (Table 1). There was no recrudescence case in Rakhine, although four cases of treatment failure were observed during the follow-up period in the 2010 study. However, 10 out of 13 treatment failures cases in Kawthaung, 6 out of 9 in Myawaddy, 1 out of 2 in Thanbyuzayat and 1 out of 6 in Shwegyin showed true recrudescence within 2009–2013 (Table 1).

### Molecular markers analysis

All of the available isolates collected on day 0 from the therapeutic efficacy studies were included for molecular analysis and a total of 550 from 6 sentinel sites were analysed for artemisinin resistance molecular marker, *k13* gene, and underlying genetic foci predisposing to become *k13* mutants, which included *pfarps10*, *pffd*, and *pfmdr2*.

### Artemisinin resistance molecular marker, K13 analysis

**Fig. 3:**
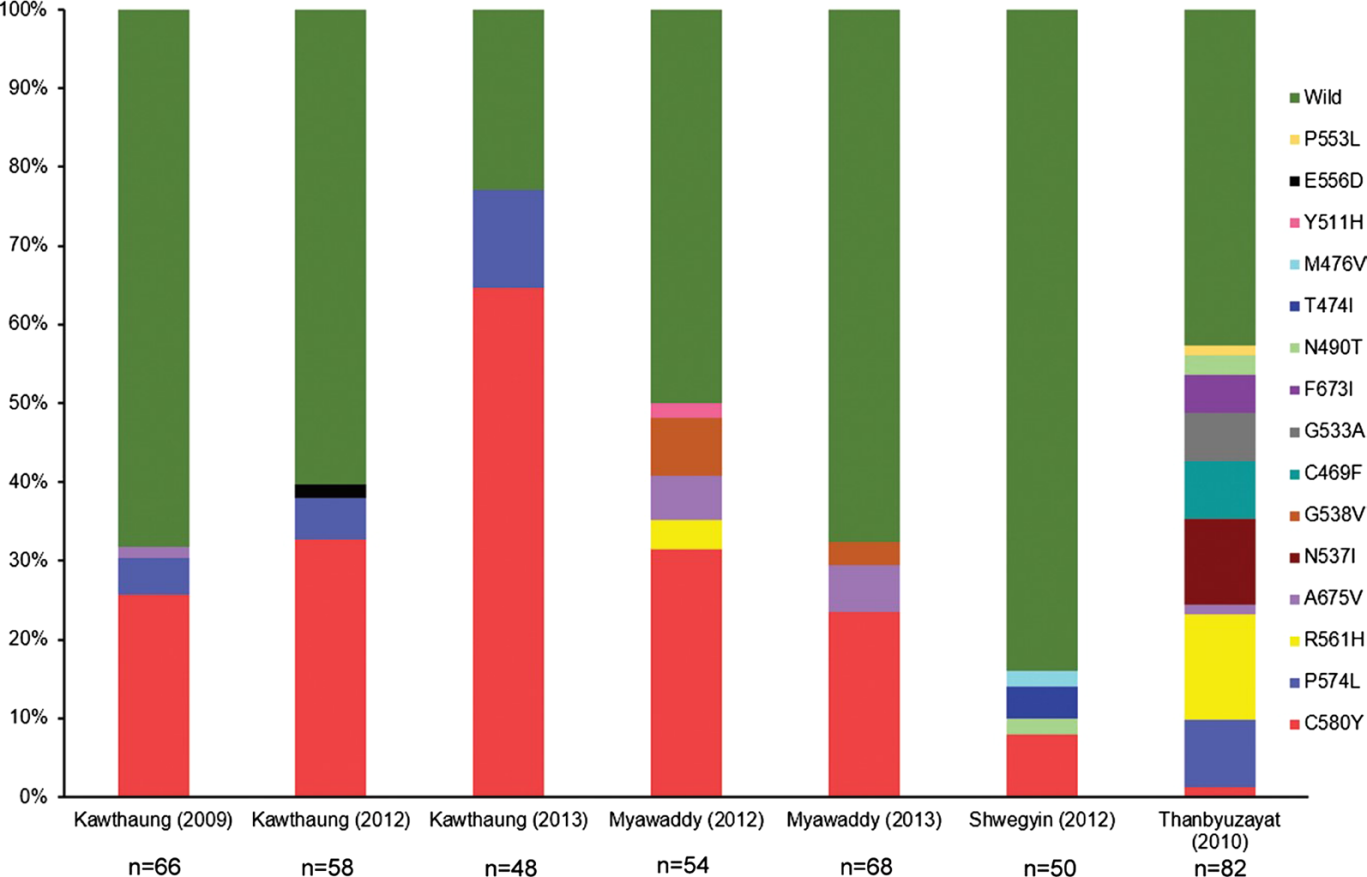
Frequency of K13 propeller alleles in 550 samples isolates in six sentinel sites in Myanmar. All mutant type isolates carry a single non-synonymous mutation. Significant decreased of wile type alleles and increasing of C580Y alleles were noted in Kawthaung, southern Myanmar site

Fifteen mutations in *k13* were observed, which accounted for 185 (33.64%) of all isolates, of which 3 mutations, E556D, F673I and M476V, were not previously reported in Myanmar. Only *k13* wild type alleles were observed in Magway and Rakhine. As shown in Fig. 3, C580Y was the predominant mutation in Kawthaung and Myawaddy while diversity of the mutations was noted in Thanbyuzayat where day 3 parasite positivity was also highest among all study sites. Moreover, both overall *k13* mutant rate and proportion of the C580Y mutation increased significantly from 2009 to 2013 in Kawthaung (*p* < 0.0001) (Fig. 3). When the association of individual *k13* mutants with day 3 positivity was assessed, C469F (*p* = 0.004), N537I (*p* = 0.004), R561H (*p* < 0.0001), C580Y (*p* < 0.004), and F673I (*p* = 0.01) were significantly higher in day 3 positivity (Table 2).

**Table 2:** Association between K13 mutations and day 3 parasite positivity

K13 mutation	Number of isolates		Total	*P*
	Day 3 negative	Day 3 positive		
T474I	2	0	2	1.000
M476V	1	0	1	1.000
C469F	2	4	6	0.004*
N490T	2	1	3	0.365
Y511H	1	0	1	1.000
G533A	4	1	5	0.531
G538V	5	1	6	0.597
N537I	4	5	9	0.004*
P553L	0	1	1	0.140
E556D	1	0	1	1.000
R561H	3	10	13	<0.0001*
P574L	14	5	19	0.166
C580Y	81	24	105	0.004^†^*
F673I	1	3	4	0.010*
A675V	9	0	9	0.621
Total	130	55	185	<0.0001^†^*

* Significantly associated K13 mutation with day 3 parasite positivity after treatment with ACT

^†^Calculated by Chi square test and all other were calculated by Fisher’s exacted test with 95% CIs

### Other molecular markers

Apart from *k13*, the sequences of *pfarps10*, *pffd* and *pfmdr2* that have been reported as underlying molecular markers predisposing to become *k13* mutant associated with delayed parasite clearance after artemisinin treatment, were also amplified and analysed: the mutation rate of these markers was found as *pfarps10* (270, 49.09%), *pffd* (322, 58.55%) and *pfmdr2* (271, 49.27%). Geographical distribution of the previously well-known artemisinin resistance molecular markers *k13* and newly reported markers, *pfarps10, pffd* and *pfmdr2* were similar in geographical distribution to that of day 3 parasite positivity after ACT in 6 different sentinel sites (Fig. 2). On the other hand, wild type prevalence of *k13*, *pfarps10*, *pffd*, and *pfmdr2* was highest in the areas where there was no day 3 parasite positivity, such as Magway and Rakhine.

### Association of molecular markers with day 3 positivity after treatment

**Fig. 4:**
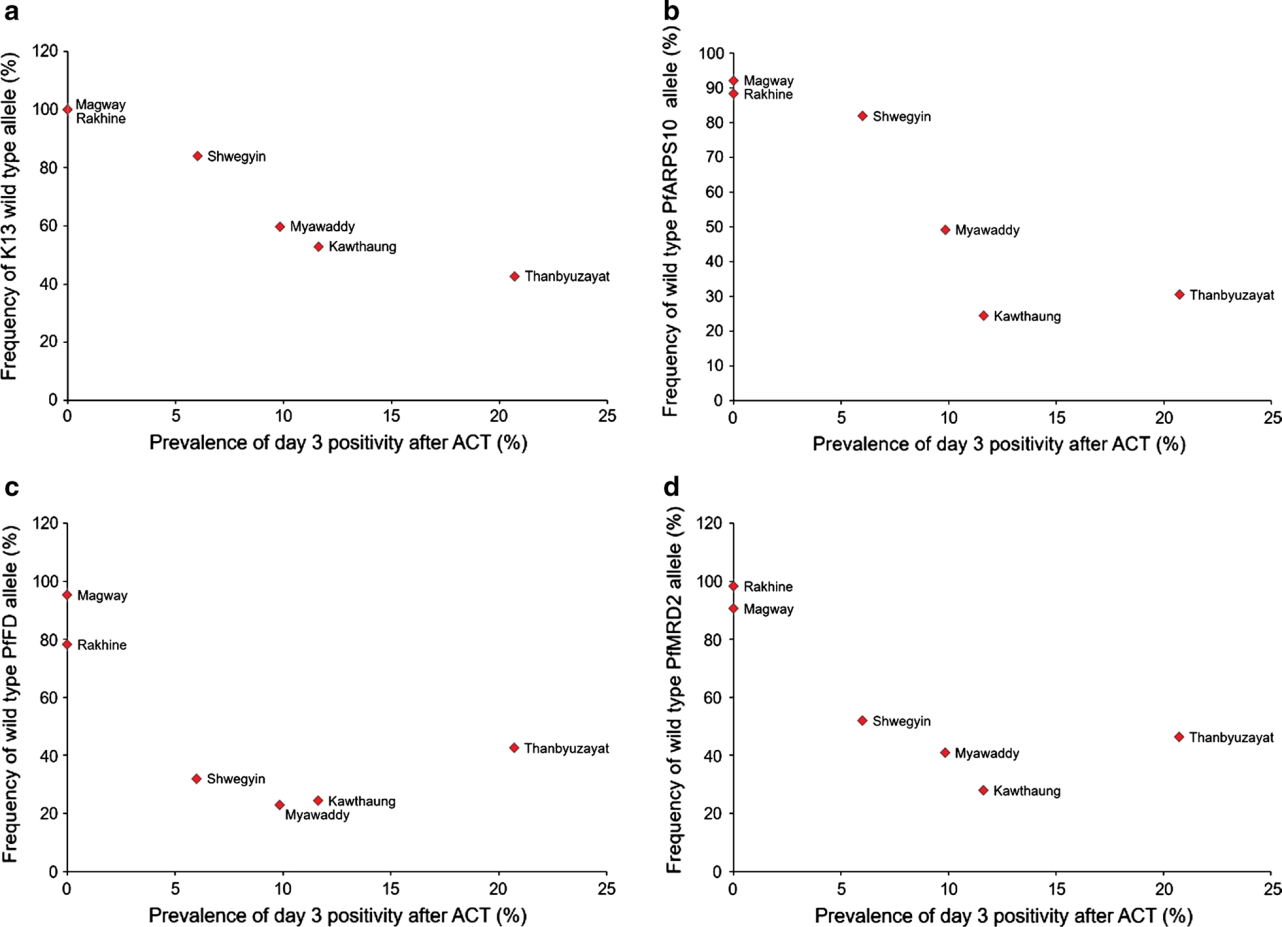
Correlation between frequencies of wile type of target genes. K13 propeller (**a**), *pfarps10* (**b**), *pffd* (**c**), and *pfmdr2* (**d**), and prevalence of day 3 parasite positivity after ACT treatment in six sentinel sites in Myanmar. The frequency of day 3 parasite positivity is plotted against the frequency of wild type alleles of target genes. Spearman’s coefficient of rank correlation: K13 propeller (*r* = −0.9590, 95% confidence interval −0.665 to −0.995); *pfarps10* (*r* = −0.8840, 95% confidence interval −0.257 to 0.987); *pffd* (*r* = −0.6704, 95% confidence interval −0.309 to −0.959) and *pfmdr2* (*r* = −0.7679, 95% confidence interval −0.115 to −0.970

The association of each target gene and combination of 2, 3 or 4 mutant alleles with day 3 parasite positivity was analysed (Table 3). Although *k13* mutant alone showed the highest odds ratio (6.48), all of the individual or combination of 2, 3 or 4 mutations were significantly associated with day 3 positivity (range of OR from 1.98 to 5.35). Regardless of *k13*, mutation in *pfarps10* alone was the highest association with day 3 positivity (OR = 3.88) followed by both mutation of *pfarps10* and *pffd* (OR = 3.28). Furthermore, when the frequency of day 3 parasite positivity is plotted against the frequency of wild type alleles of target genes, higher mutations in the targets, *k13* (*r* = −0.9590); *pfarps10* (*r* = −0.8840); *pffd* (*r* = −0.6704); and, *pfmdr2* (*r* = −0.7679) showed lower frequency of the wild type alleles (Fig. 4).

**Table 3:** Association between single nucleotide polymorphism in target genes and day 3 parasite positivity after artemisinin combination therapy

Target gene	SNP(s)	Odd ratio	95% CI	*P*
*k13*	K13^a^	6.4810	3.4095–12.3197	<0.0001
*pfarps10*	V127M	3.8841	1.9900–7.5808	<0.0001
*pffd*	D193Y	2.8784	1.4456–5.7312	0.0018
*pfmdr2*	T484I	2.5181	1.3619–4.6559	0.0025
*k13* + *pfarps10*	K13 + V127M	5.3470	2.919–9.7948	<0.0001
*k13* + *pffd*	K13 + D193Y	4.9164	2.7202–8.8855	<0.0001
*k13* + *pfmdr2*	K13 + T484I	4.4647	2.4812–8.0341	<0.0001
*pfarps10* + *pffd*	V127M + D193Y	3.2796	1.8004–5.9742	0.0001
*pfarps10* + *pfmdr2*	V127 +T484I	2.5541	1.4344–4.5478	0.0011
*pffd* + *pfmdr2*	D193Y + T484I	1.9758	1.1105–3.5152	0.0188
*k13* + *pfarps10* + *p*fmdr2	K13 + V127M + T484I	4.2245	2.3335–7.6479	<0.0001
*k13* + *pffd* + *pfmdr2*	K13 + D193Y + T484I	3.4875	1.9084–6.3731	<0.0001
*k13* + *p*farps10 + *p*ffd	K13 + V127M + D193Y	4.8354	2.6840–8.7113	<0.0001
*pfarps10* + *pffd* + *pfmdr2*	V127M + D193Y + T484I	2.2926	1.2798–4.1069	0.0044
*k13* + *pfarps10* + *pffd* + *pfmdr2*	K13 + V127 M + D193Y + T484I	3.7588	2.0371–6.9356	<0.0001

*SNP* single nucleotide polymorphism, *CI* confidence interval, *ACT* artemisinin-based combination therapy

^a^For K13, any non-synonymous mutation in kelch propeller domain, after amino acid position 440 was analysed

## Discussion

In this prospective cohort study, day 3 parasite positivity after ACT and *k13* mutants were identified in Shwegyin (southern part of central Myanmar), Kawthaung (southern Myanmar), Thanbyuzayat, and Myawaddy (southeastern Myanmar). These findings provide strong evidence that there is a high rate of *k13* mutants associated with day 3 parasite positivity after ACT had been distributed in Kawthaung, southern Myanmar, as early as 2009. Moreover, distribution of *k13* mutants and day 3 parasite positivity was similar in distribution, indicating artemisinin resistance was highest in southern and southeast Myanmar. This finding reports the usefulness of newly identified genetic markers such as *pffd*, *pfarps10* and *pfmdr2* for surveillance of artemisinin resistance, correlating with clinical follow-up data.

Emergence and spread of artemisinin-resistant falciparum malaria has been threatening the global control of malaria [14, 21]. To know anti-malarial drug efficacy and drug resistance, four main methods were available, composed of therapeutic efficacy study with longitudinal follow-up, in vitro drug sensitivity assay, molecular markers analysis, and anti-malarial drug concentration measurement [7].

Monitoring the efficacy of first-line or second-line ACT every 2 years in all falciparum-endemic countries was initiated by WHO to understand the proportion of day 3 parasite positivity after treatment and proportion of treatment failure by 28-day or 42-day follow-up [5]. If a treatment failure rate becomes more than 10%, it is time to review national anti-malarial treatment policy [22]. Day 3 parasite positivity after ACT treatment is of great concern as it exposes more parasites to the partner drug, potentially increasing the development of resistance to partner drugs [5]. In Myanmar, artemisinin-resistant falciparum malaria was recognized in 2009 and delayed parasite clearance was observed in all first-line anti-malarials [5, 7]. The overall efficacy of anti-malarials was 96.87% with day 3 parasite positivity of 8.0%. Interestingly, there was no case of day 3 persistence of parasitaemia after ACT in Magway (western part of central Myanmar) and Rakhine (western Myanmar).

Importantly, all recrudescence cases were detected in southern, southeast and the southern part of central Myanmar. All treatment failure cases reported from Rakhine, western Myanmar were re-infection. Moreover, ACPR was more than 90% in all sentinel sites (Table 1), and there was no evidence of decreasing ACPR. Unlike the Thai-Cambodia border areas where a study showed multi-drug-resistant falciparum malaria [23], there was low resistance of partner drugs such as mefloquine, piperaquine or lumefantrine in Myanmar [5]. Among the ACT, day 3 parasite positivity was highest in dihydroartemisinin–piperaquine group in Kawthaung, up to 18.75% in 2009. Similarly, dihydroartemisinin–piperaquine showed day 3 parasite positivity of 20.5% in Thanbyuzayat and 5.9% in Myawaddy site, while there was no day 3 parasite positivity in western Myanmar site. Furthermore, day 3 parasite positivity after dihydroartemisinin–piperaquine treatment in Shwegyin was 4.2% in 2009 and it increased to 12.8% in 2013 suggesting potential piperaquine resistance.

Globally, molecular marker analysis has been widely used to assess the emergence and spread of drug-resistant malaria [13, 24]. After discovery of the *k13* molecular marker for artemisinin resistance [25], many studies globally focused on the *k13* gene [13, 26, 27]. Delayed parasite clearance, as well as *k13* mutations, were reported in Cambodia [25, 28], Thailand [3, 29–31], Vietnam [32], Laos [33], southern China [34, 35], and Myanmar [9, 24]. However, more than 100 non-synonymous mutations in *k13* genes of falciparum malaria parasite were reported [5, 13, 27] and all of these non-synonymous mutations were not associated with artemisinin resistance. To the best of knowledge, only Y493H, R539T, R561H, I543T, and C580Y were accepted as validated *k13* mutations for artemisinin resistance and P441L, F446I, G449A, G538V, P553L, V568G, P574L and A675V as candidates markers [5]. Moreover, most of the *k13* mutations were reported by cross-sectional study only. Validation of common *k13* mutations by in vitro or in vivo longitudinal follow-up study to correlate delayed parasite clearance is crucial to understanding and interpreting *k13* mutations in endemic areas. In the study, C469F, N537I, R561H, C580Y, and F673I were significantly associated with day 3 parasite positivity. The mutation N537I was previously observed in eastern Myanmar [9, 10], close to the Thailand border, and also in Cambodia [25]. Similarly, C469F was previously reported in Myanmar [9, 10]. Interestingly, both of these mutations observed in the Thanbyuzayat study site were geographically close to the previously identified region, Kayin State [9, 10] suggesting the mutation was contributing to artemisinin resistance in this area. Well-known validated artemisinin resistance mutation of *k13*, C580Y has been observed predominantly in the Kawthaung study site since 2009. The prevalence of C580Y in this study site has been increasing year by year and occupied more than 60% of isolates in 2013, indicating the increasing threat of artemisinin resistance in Kawthaung. The mutation C580Y was also found predominantly in Southeast Asian countries, including Thailand, Cambodia and Vietnam [12, 30]. The mutation F446I, predominant *k13* mutation in southern China [36], northern and eastern Myanmar [10] was not observed in this study. Similarly, the mutation F673I, one of the mutations found in Southeast Asia [26] was also significantly associated with delayed parasite clearance. Evidence suggested that distinct alleles originating from independent emergence were reported rather than spread from one resistance hotspot [12], indicating the reason for occurrence of some predominant mutations in specific geographical region [27].

According to the genome-wide association study (GWAS) [17], other artemisinin resistance molecular markers were reported. In this study, the *pfarps10*, *pffd* and *pfmdr2* were studied and *pfcrt* gene was excluded because previous GWAS showed all isolates in Myanmar were mutant *pfcrt* alleles. Unlike *k13*, all of these target genes have specific non-synonymous mutations associated with delayed clearance of parasite after treatment. V127M of *pfarps,* D193Y of *pfmdr2* and T484I of *pfmdr2* were distributed in the same geographical regions as *k13* mutations [17]. In this study, prevalence of these mutations was significantly associated with day 3 positivity after treatment with ACT. Relatively fewer rates of these mutations were observed in western Myanmar where there was no case of day 3 positivity and no *k13* mutant. Moreover, individual mutations showed the association of delayed parasite clearance in *pfarps10* (OR: 3.8841, *p* < 0.0001), *pffd* (OR: 2.8784, *p* = 0.0018), *pfmdr2* (OR: 2.5181, *p* = 0.0025), indicating the suitability of markers for surveillance of artemisinin resistance. Similar findings of GWAS suggested *pfarps10*, *pffd* and *pfmdr2* polymorphism are useful markers of genetic background on which *k13* mutations are likely to appear. *K13*, as well as these underlying genetic foci, were also reported in asymptomatic falciparum infections [19] and migrant workers [20] in Myanmar. Surveillance of artemisinin resistance using *pfarps10, pffd* and *pfmdr2* should be encouraged, especially for areas where no *k13* mutant have been documented. Moreover, elimination of drug-resistant falciparum malaria should be encouraged with strong international endeavour to prevent the spread of drug-resistant malaria.

## Conclusions

K13 kelch propeller mutations and higher rate of specific mutations on *pfarps10* (V127M), *pffd* (D193Y) and *pfmdr2* (T484I) in Kawthaung (southern Myanmar), Myawaddy and Thanbyuzayat (southeast Myanmar) and Shwegyin (southern part of central Myanmar), where day 3 positivity after ACT were observed. Meanwhile, a lack of both day 3 parasite positivity and *k13* mutant, and low prevalence of specific mutation in *pffd*, *pfarps10* and *pfmdr2,* in western Myanmar indicated artemisinin resistance has not spread or emerged in these areas. Among the observed 15 mutations in *k13*, C469F, N537I, R561H, C580Y, and F673I were significantly associated with day 3 positivity after ACT. Taken together, urgent action to eliminate artemisinin-resistant parasites is needed with scaling up of regular in vivo and molecular surveillance of drug resistance, especially in western Myanmar, targeting not only *k13,* but also *pfarps10, pffd* and *pfmdr2*.
